# Barriers and facilitators of implementation of a community cardiovascular disease prevention programme in Mukono and Buikwe districts in Uganda using the Consolidated Framework for Implementation Research

**DOI:** 10.1186/s13012-020-01065-0

**Published:** 2020-12-09

**Authors:** Rawlance Ndejjo, Rhoda K. Wanyenze, Fred Nuwaha, Hilde Bastiaens, Geofrey Musinguzi

**Affiliations:** 1 Department of Disease Control and Environmental Health, School of Public Health, College of Health Sciences, Makerere University, Kampala, Uganda; 2 Department of Primary and Interdisciplinary Care, Faculty of Medicine and Health Sciences, University of Antwerp, Antwerp, Belgium

**Keywords:** Adoption, Cardiovascular disease, Community health workers, Implementation

## Abstract

**Background:**

In low- and middle-income countries, there is an increasing attention towards community approaches to deal with the growing burden of cardiovascular disease (CVD). However, few studies have explored the implementation processes of such interventions to inform their scale up and sustainability. Using the consolidated framework for implementation research (CFIR), we examined the barriers and facilitators influencing the implementation of a community CVD programme led by community health workers (CHWs) in Mukono and Buikwe districts in Uganda.

**Methods:**

This qualitative study is a process evaluation of an ongoing type II hybrid stepped wedge cluster trial guided by the CFIR. Data for this analysis were collected through regular meetings and focus group discussions (FGDs) conducted during the first cycle (6 months) of intervention implementation. A total of 20 CHWs participated in the implementation programme in 20 villages during the first cycle. Meeting reports and FGD transcripts were analysed following inductive thematic analysis with the aid of Nvivo 12.6 to generate emerging themes and sub-themes and thereafter deductive analysis was used to map themes and sub-themes onto the CFIR domains and constructs.

**Results:**

The barriers to intervention implementation were the complexity of the intervention (complexity), compatibility with community culture (culture), the lack of an enabling environment for behaviour change (patient needs and resources) and mistrust of CHWs by community members (relative priority). In addition, the low community awareness of CVD (tension for change), competing demands (other personal attributes) and unfavourable policies (external policy and incentives) impeded intervention implementation. On the other hand, facilitators of intervention implementation were availability of inputs and protective equipment (design quality and packaging), training of CHWs (Available resources), working with community structures including leaders and groups (process—opinion leaders), frequent support supervision and engagements (process—formally appointed internal implementation leaders) and access to quality health services (process—champions).

**Conclusion:**

Using the CFIR, we identified drivers of implementation success or failure for a community CVD prevention programme in a low-income context. These findings are key to inform the design of impactful, scalable and sustainable CHW programmes for non-communicable diseases prevention and control.

**Supplementary Information:**

The online version contains supplementary material available at 10.1186/s13012-020-01065-0.

Contributions to the literature

- This study highlights the implementation of a community-based cardiovascular disease prevention programme and how prior processes of assessing the needs of the community and implementers influenced implementation.
- The use of the Consolidated Framework for Implementation Research (CFIR) to understand implementation barriers and facilitators within a community setting is contextualised.
- We highlight how the CFIR domains and constructs may positively or negatively influence intervention implementation and present this diagrammatically to ease comprehension.
- The comprehensive evaluation of an ongoing implementation process and addressing challenges while leveraging on available opportunities is key to achieve impact.

## Background

Worldwide, over 40 million deaths, 71% of all global deaths, were attributed to non-communicable diseases (NCDs) in 2016 disproportionately affecting low- and middle-income countries [1]. Indeed, there is an increasing prevalence of NCDs in sub-Saharan Africa (SSA) as disease burden shifts from mostly communicable conditions [2]. The SSA region experiences over a million cardiovascular disease (CVD) deaths annually with several risk factors also on the rise [3]. For example, hypertension prevalence among persons aged 18 years and above is estimated between 24% and over 50% [4, 5]. In Uganda, over 25% of the adult population are hypertensive but awareness remains low [6, 7] and similarly is the population knowledge on CVD and related risk factors [8, 9]. Broader determinants of health such as globalisation and urbanization have had influence on population lifestyles thus contributing to the increasing prevalence and incidence of modifiable CVD risk factors [10, 11].

Countries in SSA including Uganda have not fully developed their health system capacity to deal with chronic conditions such as CVD with gaps in human resources capacity, equipment and drugs [12, 13]. Thus, to address the CVD burden, cost-effective and sustainable community-wide interventions premised on health promotion and disease prevention are the mainstay in low-income contexts [14]. These interventions should involve raising awareness on CVD, encouraging adoption of healthy lifestyles, and detecting and treating risk factors early. With this, the ability of individuals, families and communities to promote and maintain health and prevent disease and disability by their own initiative is enhanced [15]. Moreover, community approaches have been successful in supporting infectious disease control efforts and improvement of health outcomes in SSA [16, 17]. However, there is limited evidence of replication of similar efforts for NCDs even with increasing evidence of the acceptability of such programmes [18].

Understanding the barriers and facilitators of implementation of community programmes for NCD prevention and control is critical to inform disease prevention and control efforts especially in SSA to deal with the high disease burden in an efficient and sustainable manner. However, there is a paucity of studies examining the implementation processes of community NCD interventions as most have focussed on their efficacy and effectiveness [19, 20]. In China, a systematic review reported barriers to CHWs engaging in NCD prevention and control to include the lack of support such as in obtaining insurance cover or official contracts, lack of economic and healthcare resources, and high technology reliance amidst its unavailability in some areas [21]. On the other hand, an integrated health system, community trust, high quality training and community health workers’ (CHWs) capacity were facilitators [21]. The Scaling-up Packages of Interventions for Cardiovascular disease prevention in selected sites in Europe and sub-Saharan Africa (SPICES) project [22] is implementing a community CVD prevention programme in Mukono and Buikwe districts of Uganda. The programme aims to assess the effectiveness of an enhanced community approach in improving population knowledge and screening for CVD risk factors, referral and enhancing lifestyle change in a real-world setting [22]. The implementation outcomes for the SPICES programme are reach, acceptability, adoption, appropriateness, feasibility, fidelity, implementation cost, coverage and sustainability [22]. In the programme, CHWs work with existing community networks and structures to conduct CVD risk assessment and promote knowledge, improved lifestyles and cardiovascular health [22]. Specifically, the programme involves training and empowerment of CHWs to lead CVD prevention and control activities within their communities. The CHWs conduct house-to-house visits within their communities to screen for risk factors using the interheart non laboratory tool—a CVD risk assessment tool based solely on clinical history and simple physical measurements [23], provide health education and promote lifestyle change through motivational interviewing and goal-setting techniques [18, 22]. CHWs also refer high risk individuals to health facilities and follow them up afterwards in the community [22].

Prior to intervention implementation, CHWs anticipated barriers in mobilising communities, the lack of accompanying treatment services, competing interests amidst limited time, and their being required to be exemplary, and they felt that training, support supervision and experience in similar work would be facilitators [18]. On the other hand, community members were concerned about being unable to access treatment for their conditions but looked forward to sufficient information being provided to them and health services being extended nearer to them [18]. This study explored the barriers and facilitators of implementation of the community CVD prevention programme in Mukono and Buikwe districts of Uganda using the Consolidated Framework for Implementation Research (CFIR).

CFIR is a determinant framework, informed by numerous implementation models, theories and frameworks, which presents several domains hypothesized to interact in rich and complex ways to influence implementation outcomes of an intervention [24, 25]. CFIR has a total of 39 constructs/sub-constructs organised around five major domains: inner setting, outer setting, intervention characteristics, characteristics of individuals involved and process factors [24] as described in Table 1. The CFIR was applied to fully understand the implementation dynamics of a community CVD prevention programme in Mukono and Buikwe districts of Uganda so as to inform programme improvements, scale-up and sustainability in similar contexts.

**Table 1:** CFIR domains and their definitions

CFIR domain	Definition
Intervention characteristics	Features of the intervention that may affect implementation. Has eight constructs: intervention source, evidence strength and quality, relative advantage, adaptability, trialability, complexity, design quality and packaging, and cost.
Outer setting	Characteristics of the external context that might influence implementation. Has four constructs: patient needs and resources, cosmopolitanism, peer pressure, and external policy and incentives.
Inner settings	Characteristics of the organization that may influence implementation with 12 constructs. These are structural characteristics, networks and communication, culture, implementation climate (tension for change, compatibility, relative priority, organizational incentives and rewards, goals and feedback and learning climate) and readiness for implementation (leadership engagement, available resources and access to knowledge and information).
Characteristics of individuals involved	Features of implementers that influence intervention implementation with five constructs: knowledge and beliefs about the intervention, self-efficacy, individual stage of change, individual identification with organization and other personal attributes.
Process factors	Strategies and linkages that may influence implementation including planning, engaging (opinion leaders, formally appointed internal implementation leaders, champions and external change agents), executing, and reflecting and evaluating.

## Methods

### Study area

The study area consists of 20 parishes in Mukono and Buikwe districts designated to receive the SPICES project intervention within a type II hybrid stepped wedge cluster randomised trial (trial registration number: ISRCTN15848572) [22, 26]. Mukono and Buikwe districts have a population of 1,000,000 persons with a male to female ratio of approximately 1:1 [27]. In the districts, more than 70% of the population resides in rural areas engaging majorly in subsistence agriculture and fishing while others operate small businesses in trading centres within the semi-urban areas. The overall study design allows for iterative process improvements where interventions are refined before implementation is stepped up to other areas. There were four planned cycles of 6 months implementation each targeting a cluster of five parishes in a stepwise manner with activities in each parish limited to four randomly selected villages [22]. For this study, data were collected in five parishes that belonged to the first cluster of the stepped wedge.

### Study design and population

This was a qualitative study that involved process evaluation of the implementation of a community CVD prevention programme through regular meetings and focus group discussions among CHWs who are involved in implementing the intervention in the study districts. In Uganda, CHWs are referred to as village health teams and are volunteers with the ability to read and write in the local language, selected by their communities to link them with the health system [28–30]. In each parish, four CHWs each responsible for a village spearhead intervention delivery [22].

### Data collection

During intervention implementation, CHWs had bi-weekly meetings with their community-based supervisor(s) who moderated the session and took notes regarding their experiences of implementing the intervention and wrote reports to capture these in addition to their own reflections and activities. The meetings always involved CHWs reporting on their progress of intervention implementation and elucidating barriers and facilitators. During the meetings, CHWs also asked questions regarding any aspects that were not clear to them and their supervisors provided refresher trainings as necessary and followed up on any other issues. Meetings usually lasted one and a half hours and reports formed part of the data for this study. At the end of the first intervention cycle of 6 months in July 2019, five focus group discussions, one for each parish, were held with all CHWs to further elaborate on their experiences and triangulate data from reports. Meetings and discussions were conducted at the health facility where both CHWs and their supervisors usually met for their feedback meetings. An FGD guide developed based on the CFIR (see Additional file 1) and had been pretested in a similar community guided the discussions and probes used. The FGD guide was structured into the introductory sections with greetings and rapport building questions followed by questions that explored the CHW approach in intervention implementation and barriers and facilitators therein. The CHWs demographic characteristics including age, sex, education level, occupation and years working as a CHW were recorded by the note taker at the end of the discussion. The FGDs were convened for all the four CHWs in the parish covering all 20 CHWs and saturation was reached as there was no new information in the final group. The FGDs were moderated by RN (male), a research team member who was not field-based and had not any engagements with the participants, supported by a community-based supervisor who audio recorded the discussion and took notes and any non-verbal cues. Among the five community-based supervisors, all of whom were graduates with experience conducting qualitative research, three were female. The FGDs were held in *Luganda*, the local language of the area, to which both the CHWs and the study team were fluent in and lasted about an hour. The discussions provided an opportunity to probe thus enhancing understanding of forces that influence effective intervention implementation [31] while collective brainstorming of ideas, issues and solutions created a “synergistic group effect” [32].

### Data management and analysis

All audio recordings from the FGDs were transcribed verbatim and concurrently translated into English by the note taker who had expertise in both languages. The moderator later read through the transcripts to check the thoroughness of the transcription process. All study transcripts (5) and process evaluation reports (41) were exported into NVivo version 12.6 for analysis. RN and GM read the transcripts and notes several times and independently developed the initial codebooks which were discussed and unified. Coding was done inductively following the latent approach [33] identifying and examining words, phrases and sentences, some with hidden meanings that represented barriers and facilitators and coding them appropriately with new codes fitted within the codebook. At the end of the coding process, similar codes were grouped into sub-themes, and these into themes all of which were then matched with the CFIR constructs considering the domain and construct of best match. Based on transcripts and reports, the team also determined valence; whether a construct or domain majorly exerted a negative (barrier) or positive (facilitator) influence on intervention implementation or both. To illustrate, sub-themes of intervention being extensive (barrier), and its implementation being incorporated into routine activities (facilitator) formed the ‘design, complexity and adaptability of intervention’ theme. This theme fitted the CFIR domain, intervention characteristics and its constructs (complexity—for extensive intervention—and adaptability—for incorporating implementation in other activities). Selected quotations supporting themes and sub-themes have been presented to supplement the study findings. The Consolidated Criteria for Reporting Qualitative Research guidelines [34] guided reporting for this study (see Additional file 2).

## Results

### Characteristics of community health workers

A total of 20 CHWs, 13 of whom were female, engaged in intervention implementation in 20 villages across the 5 parishes. The average age of the CHWs was 49 years (range 34–65 years) with over a half (13/20) aged between 34 and 50 years and the rest above 50 years. Eleven CHWs had attained secondary education and others received only primary education. Almost all CHWs (18/20) engaged in farming for subsistence purposes and over half (11/20) had served their communities for more than 15 years.

### Barriers and facilitators

**Fig. 1:**
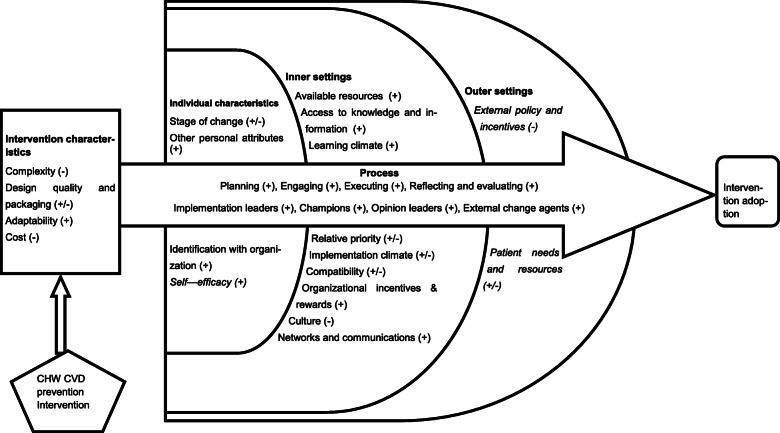
CFIR constructs and their influence on implementation of a community CVD prevention intervention

Drivers of intervention implementation success or failure which spanned 26 of CFIR’s 39 constructs were identified as illustrated in Fig. 1. Of these constructs, 4 were majorly barriers, 16 facilitators and 6 both barriers and facilitators. Emerging themes from analysis of transcripts are presented under the CFIR major domains embedding the framework constructs except for the process domain constructs that have been integrated within the others (intervention characteristics, outer settings, inner settings and characteristics of individuals involved). The details of themes, sub-themes and supporting participant quotations are presented in detail below and summarized in Table 2.

**Table 2:** Summary of themes and sub themes highlighting barriers and facilitators of CHW CVD prevention intervention implementation

CFIR domain and constructs	Theme	Sub-theme (barriers)	Sub-theme (facilitators)
Intervention characteristics ▪ Design quality and packaging ▪ Complexity ▪ Adaptability ▪ Cost	Design, complexity and adaptability of intervention	• Intervention is extensive • Difficulties with filling forms and doing calculations • Intervention activities time consuming • Behaviour change is not easy • Not finding men at home during their visits and fishing communities being mobile	• Incorporated the intervention within other routine activities • Focussed education majorly on risks identified during the interheart screening • Educated family members together on general risks before individual counselling • Utilised public gatherings to supplement house visits which were also done on evenings and weekends
	Quality and supply of inputs	• Waist and hip ratio tape measure breaking down. • Calculators not provided for calculation of waist and hip measures and adding interheart scores.	• Waist and hip ratio tape measures replaced with those of better quality. • CHWs used phones were available.
	Gradual change process	• Community behaviour change is slow.	• Encouraged incorporation of lifestyle practices into daily routines. • Elaborated cost of unhealthy behaviours. • Utilised motivational interviewing techniques. • CHWs shared experiences among themselves.
	Costs of fieldwork	• Large distances due to big sparsely populated villages • Unfavourable weather • Doing fieldwork while sometimes hungry • Less time for other responsibilities	• Some CHWs had smaller villages easing field work • CHWs provided with gumboots and umbrellas to help during harsh weather • Planning time and going to the field in the afternoon after lunch.
Outer settings ▪ External Policy and Incentives ▪ Patient needs and resources	Resources availability,	• Community demands: a playing field and balls to increase their physical activity levels, blood pressure machines to measure blood pressure at home and drugs for treatment, fruits and vegetables and their seedlings to increase supply.	• Encouraged community members to start vegetable gardens • Provided community members with their own seedlings where possible • Encouraged community members to seek care from health facilities which had been strengthened • Liaised with health workers to conduct outreaches
	Health services accessibility and quality,	• Unavailability of required services or their being of poor quality. • Health worker negative about CHW referral	• Availability of quality health services. • Health worker positive about CHW referral • Health workers involvement in CHW training • Transfer of health workers
	Media reinforcement		• Media raised awareness on CVDs and reinforced messages passed by CHWs • Media message consistency with that passed by CHWs.
	Policies and procedures.	• Non remuneration of CHWs • Prioritising existing CHWs for community engagements	• Replacement of some CHWs engaged with many activities to devote time to the intervention.
Inner settings ▪ Available resources ▪ Access to knowledge and information ▪ Learning climate ▪ Tension for change ▪ Relative priority ▪ Implementation climate ▪ Compatibility ▪ Organizational incentives and rewards ▪ Culture ▪ Networks and communications	Training and learning environment		• CHW training on intervention and its implementation including piloting field work. • Presence of training manuals in local language for consultation. • Positive learning environment.
	Community awareness and interest	• Low awareness and perceived risk of CVDs. • Uncooperative members and access barriers.	• High awareness and perceived risk of CVDs. • CVD screening programmes. • Use of community strictures such as leaders and groups. • Encouraging group activities such as for physical exercise.
	Trust	• Mistrust of CHW motives attributed to politics or western interests	• Trust of CHWs. • CHW popularity and close relationship with community. • Local leaders support • Project branded t-shorts eased identification with community members.
	Culture and beliefs	• Unease in taking waist and hip measurements of opposite genders. • Wrong perception of the need for waist and hip measurements. • Physical activity related activities such as running or riding a bicycle was not culturally acceptable. • Tendency to cook only one kind of food without balancing diet. • Belief that fruits and vegetables are meant for young children and sometimes sold for income.	• Carried out the measurements in public places while adopting a sideways posture. • Requested a family member to support taking measurements • CHWs providing thorough explanations to community members regarding need for measurements
	Demographic composition	• Resistance, several questions and less cooperation among youths and males.	• Elderly and female community members more cooperative.
	Support supervision and feedback		• Frequent support supervision and feedback • Setting and reviewing goals and targets • Continuous refresher training for CHWs • Addressing CHW feedback and providing response • Friendly and approachable supervisors who communicated well.
Characteristics of individuals involved ▪ Individual stage of change ▪ Other personal attributes ▪ Individual identification with organization ▪ Self-efficacy	Stage of change	• Lower spectrum to stage of change	• Higher motivation to change such as those already hypertensive or diabetic. • Community members’ testimonies. • CHW experiences and exemplariness.
	Competing demands	• High workload due to several CHW work tasks and other personal responsibilities	• Set aside time for intervention implementation. • Incorporate intervention duties within similar usual works. • Utilise community engagements for example at public events to share intervention message. • Setting targets and goals bi-weekly. • Flexibility in scheduling the meetings.
	Motivation and commitment	• Lack of financial incentives	• Motivation from non-financial sources including the recognition and respect and project incentives such as t-shirts and training certificate. • Observed changes in community behaviours and reported improved health outcomes. • Transport refunds for the bi-weekly meetings.
	CHW attributes	• Being village leaders ensured that CHWs were busy to devote sufficient time to intervention implementation. • Low experience in dealing with community. • CHW sickness	• Some CHWs were village leaders having influence and authority. • CHWs supporting referral at health facility. • High CHW self-efficacy and experience
	Socio-demographic characteristics	• Older and female CHWs found it harder to influence the youth and male community members	• higher educated CHWs grasped concepts much faster, explained them better and produced data of good quality • Personal experiences of CHWs who had CVD risk factors.

### Intervention characteristics

Under this domain, four themes emerged: design, complexity and adaptability of intervention; quality and supply of inputs; gradual change process and costs of fieldwork. These themes embedded four CFIR constructs of design quality and packaging, complexity, adaptability and cost.

#### Design, complexity and adaptability of intervention

The level of complexity and flexibility of the intervention was a key factor in its implementation. CHWs sometimes expressed concern that the intervention was extensive with elements of risk factor screening using interheart, goal setting and motivational interviewing and referral and follow-up (complexity). CHWs also sometimes reported difficulties with filling the interheart form especially explaining questions on stress and depression, calculating the waist and hip ratios and compiling the interheart scores (complexity). The intervention activities were also noted to be time-consuming as community members asked many questions especially the youths and elderly members and CHWs would only reach a few households while in the field. CHWs also stated that behaviour change was not easy adding to the complexity.

*“I teach individuals the different risk factors for CVDs. I teach about the health risk of excess body weight and I measure their waist and hip circumference and calculate their score to ascertain if they are overweight. I thus take a lot of time working on one person and by the time I finish administering the interheart form, taking the waist-hip circumference measurements and adding up the scores, I get no time to work on other things and I don’t get to visit many households.”* [FGD 4, CHW 4]

To cope, CHWs usually incorporated routine activities within the CVD intervention delivery such as inspecting household sanitation after CVD risk factor screening and education. Moreover, CHWs focussed their education majorly on risks identified during the interheart screening and educated family members together on general risks before providing individual counselling or planned community-wide events and utilised public gatherings to supplement house visits (adaptability). These measures led to efficient use of time and eased intervention progress.

#### Quality and supply of inputs

The provision and regular supply of intervention inputs including interheart and referral forms, information sheets for community members and waist and hip tape measures was key in intervention implementation. CHWs mentioned that the forms were provided in the local language which they preferred, were simple to understand and use (design quality and packaging). This notwithstanding, in rare instances, some community members who were not fluent in the local language requested for forms in English (complexity) which were later provided. The CHWs noted that health workers too preferred referral forms in English as opposed to the local language forms that had been provided to the CHWs. However, CHWs countered this demand from health workers who understood that the local language was much easier for the CHWs (process—engaging). The CHWs were provided waist and hip ratio tape measures for use during the intervention which sometimes broke down (design quality and packaging) but were often replaced with those of better quality (cost). The CHWs sometimes required calculators (design quality and packaging) for their calculations of waist and hip ratios and adding up interheart scores but these were not provided and resorted to using mobile phones where available (adaptability).

*“I got some challenge, my waist-hip ratio measuring tape was weak and got damaged while in the field, but they brought me another one which is of better quality that I am currently using.”* [FGD 5, CHW 1]

#### Gradual change process

Local leaders, CHWs and the community believed that the intervention created a difference (relative advantage) based on experiences of fellow community members and the consistency of message they had heard over the years through health workers and the media (evidence strength and quality). CHWs though noted that behaviour change of community members was slow (complexity). To elicit change, CHWs therefore encouraged community members to incorporate lifestyle practices into their daily routines such as farming to achieve their physical activity goals (adaptability), often elaborated the cost of unhealthy behaviours to community members such as funds spent on alcohol which usually proved eye-opening. CHWs also utilised motivational interviewing techniques encouraging CHWs to undergo a gradual process of behaviour change such as setting goals to reduce number of cigarettes smoked in addition to continuous health information sharing and follow-up (cost). Additionally, CHWs continually shared experiences amongst themselves to foster learning and sometimes worked as a team to support one another (process—champions).

*“When we computed the amount he was spending on alcohol in a year, he was alarmed and exclaimed, ‘My God! I have never had such money in a whole year.’ I advised him to start reducing his consumption from six sachets to four and save money for two sachets and later reduce further. He left convinced that he was wasting a lot of money.”* [FGD 4, CHW 2]

#### Costs of fieldwork

The implementation strategy involved CHWs traversing their whole village to visit all households therein. However, some villages were large and sparsely populated and thus CHWs had to move large distances to reach households usually through dusty, rough and uneven terrain, harder to navigate during the rainy season (cost). CHWs were provided with protective wear including gumboots and umbrellas to support them during the rainy season (cost). Relatedly, CHWs incurred opportunity costs especially in reduced time to fulfil other social and gender responsibilities such as parenting, joining social gatherings or attending to their other work (cost).

### Outer settings

Within the outer setting domain, four themes emerged: resources availability, health services accessibility and quality, media reinforcement and policies and procedures. These themes fitted two CFIR constructs: external policy and incentives and patient needs and resources.

#### Resources availability

Availability of resources to accommodate the promoted behaviour changes in the community was a barrier in intervention implementation. For instance, community members demanded for a playing field and balls to increase their physical activity levels, blood pressure machines to measure their blood pressure at home and drugs for treatment (patient needs and resources). Other resources of interest to the community were fruits and vegetables and their seedlings as these were limited in most areas owing to their seasonal nature (patient needs and resources). CHWs in these instances encouraged community members to start vegetable gardens within small spaces that they can irrigate and encouraged community members to seek care from health facilities which had been strengthened by the SPICES project (process—external change agents). Sometimes CHWs liaised with health workers to extend CVD screening services to the community which was well received (process—engaging, external change agents).

#### Health services accessibility and quality

The availability, accessibility and quality of health services were key determinants of intervention uptake. Where CVD services were available, accessible and considered of a high quality, community members expressed willingness to interact with the community intervention and where necessary sought more support from the health facility (patient needs and resources). Community members’ testimonies encouraged other members to go to the facilities easing the CHW work (process—champions).

*“As I told you, the people we go to are happy and they like the [SPICES] programme. When a person you have worked on goes to the health facility and they are well attended to and given drugs, they go on sharing this information. Later, you see people from other households that you have not yet reached asking you: ‘Doctor, why did you abandon us?’, You tell them that ‘I have not abandoned you and I will be coming to your place soon’. People are happy and they like the services.”* [FGD 5, CHW 1]

On the other hand, where referred community members are not attended to or do not receive adequate services, their testimony became a barrier to CHW work (patient needs and resources). Also, how health workers received and interacted with referrals from CHWs mattered with those who were positive further motivating CHWs in referring more individuals unlike those who under looked their referrals (process—external change agents). Health workers at facilities were also involved in the CHW training and disseminations (external policy and incentives) to bridge the gap between the two and create rapport for future interactions and referrals that CHWs appreciated (process—engaging). It was also much easier for CHWs to sometimes reach out to health workers if they had any questions or to plan community activities (process—engaging). However, sometimes health workers were transferred (external policy and incentives) and CHWs had to build rapport afresh at the facility slowing their work (process—engaging).

#### Media reinforcement

The media (process—external change agents) also played a role in intervention implementation especially where it raised awareness on CVDs and reinforced messages that had been passed by CHWs (external policy and incentives). In fact, consistency of messages was key in facilitating the intervention because where community members received a similar message from the CHWs, at the health facilities and from the media, it increased their trust in CHWs and eagerness to comply with their messages (process—external change agents).

#### Policies and procedures

The intervention was planned and executed within available policies and procedures including those around remuneration where CHWs are expected to be voluntary and are not paid any regular emoluments (external policy and incentives). This sometimes led to dissatisfaction and low motivation of CHWs. The policy that pre-existing CHWs should be given priority in community programmes was challenging especially where some CHWs were engaged in multiple activities with little time devoted to the intervention and a suboptimal performance which warranted replacements (external policy and incentives).

### Inner settings

The emerging themes within the inner settings were training and learning environment, community awareness and interest, trust, culture and beliefs, demographic composition, support supervision and feedback. These themes reflected 10 CFIR constructs: available resources, access to knowledge and information, learning climate, tension for change, relative priority, implementation climate, compatibility, organizational incentives and rewards, culture and networks and communications.

#### Training and learning environment

Before intervention implementation, CHWs underwent a five half-day face to face training which included theoretical and practical aspects of the intervention and the roles and responsibilities they were expected to play, and a two-day field orientation (available resources). This training and dissemination events were a facilitator of intervention implementation and many CHWs noted that it empowered them with the knowledge, skills and confidence (organizational incentive and reward) and provided them with learning materials such as manuals that they continuously consulted (access to knowledge and information). The provided equipment and non-financial incentives such as t-shirts, gumboots and umbrellas (available resources) were also facilitators. The other aspects of the training they appreciated were the simplification of materials in the local language for ease of understanding (access to knowledge and information), the piloting field works that reinforced learning and the positive learning environment where all their questions were answered freely (learning climate).

#### Community awareness and interest

The awareness and interest of community members in CVD prevention activities played a role in intervention implementation. In fact, community members who were more aware about CVDs or thought of it as a big problem (tension for change) were more cooperative with CHWs and honoured referrals compared to those with low levels of awareness who usually had a low perceived CVD risk and were reluctant to seek healthcare (relative priority). In fact, screening programmes played a big part in increasing awareness of CVD risk factors and stimulated community participation in the project (process—engaging). Sometimes community members felt that other conditions were more urgent than the CVDs and would divert the CHW to other diseases such as malaria during house visits or education (relative priority). In some urban areas, CHWs were sometimes met with walled fences and uncooperative members who would not let them in their gated houses making access to these households much harder (implementation climate, culture).

Existing community structures were utilised (compatibility, process—engaging) in order to increase community awareness, buy-in and interest. These structures included community leaders such as local and religious leaders (process—opinion leaders), community groups such as savings groups, and community events such as meetings (process—executing). Where community structures were involved, CHWs felt more support and intervention implementation moved more smoothly. For example, community leaders supported mobilisation of their communities and the church leaders identified with the intervention usually inviting CHWs to share key messages during their gatherings (process—champions). For savings groups especially those to which CHWs belonged, they were usually allocated time after their usual meetings to talk about the intervention (process—executing). CHWs also encouraged group activities especially for physical activity mobilising community members to meet and exercise regularly which was more appealing especially to the youths (process—engaging).

*“The CHW mobilised the local council committee members of his village to take part in three sessions of health education on CVD prevention and control. The local council chairperson welcomed the project and its intentions and pledged to mobilize all his committee members for the education sessions and support the project. The first, second and third sessions were planned and took place in June and July attracting several community members.”* [CHW supervisor report]

#### Trust

Although most community members trusted the CHWs and this fully facilitated intervention implementation (compatibility), there were instances of mistrust where members attributed CHW activities to politics or western interests which sometimes hindered cooperation (relative priority). CHWs relied on their popularity and close relationship with their community having worked or lived in the area or their ability to build rapport (networks and communications) and sometimes sought help (process—engaging) from local leaders (process—opinion leaders) to provide re-assurances. Moreover, branded project t-shirts (organizational incentives and rewards) also eased their identification to community members (process—formally appointed internal implementation leaders).

*“Some don’t want to tell us their age or about themselves. They see us as spies and they say, ‘why are you asking me all this, are you a spy? They usually don’t like so many questions. As you reply, you must handle them with politely. You can explain to them that I am a fellow community member, you cannot suspect that I can have bad intentions towards you. Then you go on to explain to them carefully that you are not a spy and that you are only interested in health issues. Then you sensitize them. Some of the community members are witnesses that what we have sensitized them about are good issues and they have worked for them.”* [FGD 2, CHW 2]

#### Culture and beliefs

One of the CHW roles was to take waist and hip measurements of community members as part of required information for filling in the interheart forms, and this aspect was sometimes considered not culturally appropriate where opposite genders were involved for potential of it being misconstrued (culture). This sometimes led to CHWs preferentially choosing community members of their gender during house visits (compatibility). Thus, CHWs sometimes carried out the measurements in public places while adopting a sideways posture and other times requested a family member to support taking measurements such as husband taking measurements of their wife (learning climate).

*“When I disapproved of her repeatedly working mostly on women, the CHW said she was uncomfortable fastening a tape measure around men as it would look like they are being touched to evoke sexual feelings. She also noted that this form of act was disapproved of in the Kiganda [local] culture. I advised her to explain to them [men] why she needs to take their waist and hip measures and where necessary solicit the support of another family member to support taking the measurements.”* [CHW Supervisor report]

CHWs said that sometimes community members interpreted the purpose of the waist and hip measurement to be intended to provide them clothing (relative priority). CHWs endeavoured to provide thorough explanations to community members regarding the intervention to wade off any suspicions and expectations (networks and communications). Some community members mentioned that running or riding a bicycle was not acceptable especially for women and thus they could not engage in such activities (culture). Regarding food choices, the community also had a tendency of cooking just one type of food such as sweet potatoes served as a big heap on one’s plate without balancing the diet and others claimed that food that is not fried is not delicious (culture). Other community members had the belief that fruits and vegetables were meant for young children or the rich while others usually looked at them as a source of income and sold them to others (culture). The CHW had to address these issues through thorough sensitisations (networks and communications).

*“Some [community members] continue asking why do you measure my buttocks and waist, are you going to buy me a trouser? One lady asked me why I did not measure her skirt to the bottom. The lady said a skirt starts from the waist to the bottom, take all measurements, and I told her no, am not measuring you for an outfit. There has been a lot of questions in that area too.”* [FGD 3, CHW 3]

#### Demographic composition

Furthermore, owing to the design of the intervention which involved house-to-house visits, CHWs sometimes did not find men at home during their visits since most worked during daytime and fishing communities were usually mobile (compatibility). Community groups also reacted differently to the intervention with CHWs reporting more resistance, several questions and less cooperation among youths and males (implementation climate, compatibility) compared to the elderly and females due to less prioritization of the intervention (relative priority, compatibility). The CHW demographic could have mediated this relationship (networks and communications) that whereas youth were more likely educated and asked teasing questions, the elderly were more interested in learning and exploring several dimensions of CVDs with CHWs. CHWs continued engaging all community groups (process—executing) and answering any questions and concerns they had (process—engaging). The level of cooperation of the community (organizational incentives and rewards) played a role in CHW motivation.

*“The CHW noted that capturing men was still a challenge, citing that most men always tell her that such programmes are for women and in any case, interviews and other data can equally be best provided by their wives. She stressed that you can find both the wife and her husband, and the husband tells you directly: ‘that is for women, I even don’t have time; you ask, screen and discuss anything with my wife’. On the other hand, some individuals claim that they don’t have any signs and symptoms of being unwell especially the youth.”* [CHW Supervisor report]

#### Support supervision and feedback

Frequent support supervision and feedback (goals and feedback) provided by community-based supervisors was key in further supporting CHWs to carry out their roles (process—formally appointed internal implementation leaders). Indeed, CHWs mentioned that working with their supervisors at the start built their confidence to deal with community members and learn how to approach questions (available resources). Supervisors continually monitored and supervised CHW activities (goals and feedback), mobilised them for fortnightly feedback meetings where any goals and targets were set, and previous ones reviewed (goals and feedback), and provided any continuous refresher training on concepts that were challenging (available resources). The feedback that CHWs got from the field work and review of filled forms enabled them to continually improve and perform better (goals and feedback). The other important component was the feedback from CHWs regarding the work they were doing (goals and feedback), and CHWs found it important when the feedback they provided to their supervisors was acted upon and a response provided to them as they felt more valued (organizational incentives and rewards). The supervisors were also noted to be friendly, approachable, communicated well and established a good relationship with CHWs (networks and communications).

*“The bi-weekly feedback meetings give us a platform to ask about things we do not know. Even after training, someone can keep at the same level of knowledge. However, during the meetings we get enough time to discuss and learn more rather than having phone calls when things might not be explained in detail..”* [FGD 1, CHW 2]

### Characteristics of individuals involved

There were five emerging themes under the individual characteristic’s domain: stage of change, competing demands, motivation and commitment, and CHW attributes. These themes were related to four CFIR constructs: individual stage of change, other personal attributes, individual identification with organization and self-efficacy.

#### Stage of change

The community member’s stage of change was a key implementation factor with those on the lower spectrum (e.g. precontemplation) sometimes resistant to receiving information and advice regardless of their practices (individual stage of change). On the other hand, individuals who had a higher motivation to change such as those who were already hypertensive or diabetic were much more open to advise and engagement with CHWs (individual stage of change) and shared valuable testimonies that attracted the attention of other community members (process—champions). CHWs (process—opinion leaders) also shared experiences among themselves to support each other and other personal experiences to encourage change including lifestyle changes made while others relied on their being exemplary to motivate community members (individual stage of change, process—engaging).

#### Competing demands

CHWs had many competing demands and priorities related to other tasks they were supposed to carry out, other personal work such as businesses and engagements including those within the community such as attending community events and visiting their relatives and friends (other personal attributes). These demands culminated into a high workload and would sometimes take up CHWs’ time and limit their engagement in intervention delivery (process—executing). CHWs were encouraged to set aside a few hours every week to make progress (process—planning) with the intervention and sometimes incorporate intervention duties within similar usual works, and this supported intervention implementation (process—executing). CHWs also sometimes creatively devised ways to still share about the intervention and pass some messages during their community engagements for example at public events (process—champions). The other key facilitator was setting targets and goals bi-weekly which stimulated CHWs to work hard and meet them for reporting during their feedback meetings and this kept them on track (process—reflecting and evaluating). There was however need for flexibility in scheduling the meetings to cater for the CHW competing demands.

*“I had scheduled to meet the CHWs but one of them called me early on the meeting day that he had a very urgent matter to attend to and would not make it for the meeting. We thus re-scheduled the meeting to another day, but this was inconveniencing to all of us. Although the meeting later took place, another CHW still left earlier to attend to other prior arranged commitments.”* [CHW Supervisor report]

#### Motivation and commitment

CHW motivation was a key factor that impacted intervention implementation. CHWs majorly derived their motivation from non-financial sources including the recognition and respect they obtained from the community and incentives provided by the project (other personal attributes). Moreover, the personal knowledge they acquired through trainings, training certificates and invitation to attend project events such as dissemination events motivated them (other personal attributes).

*“Attending the project dissemination event uplifted and gave us some bit of change. It helped us analyse our performance because at first, we just worked for the sake but when we got to meet other CHWs and saw how they performed, it gave us more passion for what we do. I really felt so challenged that immediately we got back from that event, I started to work such that we can reach the level of the others.”* [FGD 2, CHW 1]

The other motivation CHWs had was from observing changes in behaviour in their villages and obtaining good feedback from community members about the intervention with some reporting improved health outcomes. Where CHWs were motivated, they showed high commitment to their work and their productivity was high (individual identification with organization). This notwithstanding, CHWs continued to demand for financial incentives and the lack of these demotivated some of them. The only financial incentive the project provided was in form of transport refunds for the bi-weekly meetings and other events which the CHWs appreciated (individual identification with organization).

*“On the same note, one CHW stated that she lacked prior motivation of doing the project’s field work because she was unsure of her attached benefit. However, she was contented with the transport refund offered during their work review meetings.”* [CHW supervisor report]

#### CHW attributes

Some CHWs were village leaders and this facilitated intervention implementation as they were more recognised and given much respect and the community were more likely to listen to them (process—opinion leaders).

*“I am the Chairperson of my village so whenever I get time, I visit a household and I share the knowledge I get from my trainers here and when I call for meetings I take about 10 minutes and briefly share with them about the project. I also get invited to savings groups cooperatives as a special guest and usually pass on messages about the project at such fora.”* [FGD 3, CHW 2]

On the other hand, CHWs who were leaders tended to be busier than their counterparts as they had to balance leadership responsibilities and CHW roles and in most cases prioritised the leadership thus dragging intervention implementation. Similarly, CHWs who also supported their health facilities usually helped the community members referred from the community to quickly access health care when they were present at the facility (process—champions). However, this too came with an increased workload and less time for community intervention implementation. The other relevant personal attribute was the CHW self-efficacy and experience doing community health work with more experienced CHWs being famous with wide networks and skilled in dealing with community members facilitating intervention delivery (self-efficacy). On the other hand, those that had limited experience required empowerment to build their confidence to assert themselves in carrying out the intervention (self-efficacy). The major personal limitation for CHWs was their health where some would take some time off due to ill health and other life responsibilities.

*“When I inquired from the CHW why she had not performed well in her village, she explained that she had episodes of sickness and was too ill to conduct home visits. In addition, she was having stress mobilising school fees for four of her children in boarding school and all these challenges impeded her work.”* [CHW supervisor report]

#### Socio-demographic characteristics

The socio-demographic characteristics of the CHW such as their age, sex and education level influenced intervention implementation (other personal attributes). Older and female CHWs found it harder to influence the youth and male community members respectively and vice versa.

*“Sometimes you find when someone is a smoker and you explain to them about the dangers of the habit and they tell you that: ‘You who was born yesterday, how can you tell me about smoking yet I have smoked for the last forty or fifty years’. Then you may just end up referring him/her to the facility for further support.”* [FGD 5, Participant 1]

On the other hand, higher educated CHWs grasped concepts much faster, explained them better and produced data of good quality (other personal attributes). CHWs who had CVD risk factors such as hypertension or diabetes relied on their personal experiences with the disease to elicit change and create interest (knowledge and beliefs about the intervention, process—opinion leaders).

## Discussion

This study explored the barriers and facilitators of implementation of a community CVD prevention programme in Mukono and Buikwe districts in Uganda using the CFIR. The framework enabled the systematic and comprehensive identification of drivers of implementation success or failure across its domains to inform and improve intervention implementation for impact and scale-up. The barriers to intervention implementation were the complexity of the intervention leading to high opportunity costs, some aspects of the intervention not being compatible with community culture, the lack of an enabling environment for behaviour change and community members sometimes mistrusting CHWs. Moreover, the low community awareness of CVD, CHW factors such as their demographics and competing demands and unfavourable policies impeded intervention implementation. On the other hand, the intervention was facilitated by availability of inputs and protective equipment which also acted as incentives, adequate training of CHWs, working with community structures including leaders and groups, frequent support supervision and engagements, CHW attributes such as motivation and commitment and access to good quality health services. These barriers and facilitators are in line with those that had been anticipated by the community and CHWs at the start of the intervention [18], those reported by a systematic review in China [21] and experiences from Bangladesh, China, Nepal and Viet Nam [35].

In implementation of a multi-component community CVD prevention programme, the complexity of the intervention needs careful consideration especially because CHWs are lay persons with no specialised CVD knowledge and skills and usually have low literacy levels [28–30, 36]. Moreover, the more complex an intervention is, the higher the opportunity cost to CHWs especially in terms of time and resources which may impact intervention fidelity [37]. Therefore, in addition to context, designed interventions should be adapted to CHW’s abilities to enhance intervention ownership, acceptability and success. Potential adjustments could include omitting certain intervention components or reducing their duration, simplifying implementation tools including those for training or education or setting manageable targets. However, where complex interventions are involved, a selection criterion that requires a higher education level would help to increase efficiency and impact. Indeed, higher education levels are associated with improved knowledge and performance among CHWs [38, 39] though they may come with higher attrition levels [40] and thus should be considered cautiously. Uganda’s CHW is voluntary with no remuneration [28–30] and CHWs continue to face other competing demands due to personal and social responsibilities [18, 41]. It is thus key that costs for CHWs are minimised as much as possible such as through providing transportation mechanisms. Moreover, in recruiting CHWs to support such demanding interventions, their other commitments need to be realistically examined to avoid those with many responsibilities who may not create time for the intervention. Beyond workload, CHW self-efficacy, motivation and commitment are key attributes that should be explored a priori during recruitment in line with the World Health Organization (WHO) guidelines on health policy and system support to optimise CHW programmes [40]. Motivated CHWs are more likely to be performers and invest time in supporting intervention implementation [38, 42] though motivation avenues should be continually incorporated within programmes. During intervention implementation, CHWs also reported some forms of mistrust by community members which although had been anticipated [18] is a concern for the success of community interventions. Mistrust could be related to the low community awareness on CVD and its risk factors [8] hence the low relative priority some members attached to it. Trust and respect are cornerstones of community health work [40, 42–44] and have been reported as a facilitator for CHW engagement in NCD prevention programmes [21, 35]. Overall, increasing community CVD awareness, relative priority and community ownership of programmes and support of the local structures would go a long way in increasing trust of CHWs [41–44].

Culture in terms of community attitudes and beliefs is another key factor that can bar or skew intervention implementation if not well managed. It is thus important that the compatibility of an intervention with community culture is ascertained in advance. In our pre implementation study, some of the discussed aspects of the intervention were not very specific to elicit precise feedback on the cultural aspects [18]. Trainings should pay close attention to any cultural aspects that may affect intervention implementation and prepare CHWs to effectively deal with them. If left unattended, undesirable consequences may arise for example CHWs finding their own coping mechanisms such as selectively dealing with community members of the same gender. Indeed, previous literature has shown that sometimes CHWs find it easier to deal with community members of their gender and similarly do community members [45] and since CHW programmes are usually dominated by females [36, 46], males—who rank higher in certain lifestyle practices such as smoking and alcoholism—may not be fully impacted. Similarly, we found the CHW demographic attributes to be an important factor in who they reach out to. Community groups such as males and youths were generally considered unwelcoming compared to females and the elderly which could largely be dependent on the demographic of the CHWs themselves, usually female and older. This reiterates the need for diversity of CHWs for effective intervention implementation such that no groups are left behind [45]. However, the WHO guidelines on health policy and system support to optimise CHW programmes downplay the significance of factors such as age and gender as selection criteria for CHWs as they may promote unfair discrimination [40]. Moreover, it may be perceived that CHWs are meant to attend to members of their gender thus creating unintended consequences. More proactive measures such as thorough training and empowerment of CHWs with skills to deal with the different groups and manoeuvre-related cultural issues would be desirable. The WHO guidelines however strongly recommend the need to remunerate CHWs based on their training, duties and roles [40] which is still an impediment in Uganda’s CHW programme [28, 29] and requires careful consideration in future programmes. Another external policy factor is the need to create an enabling environment for the practice of healthy lifestyle factors. Sustainable initiatives such as the promotion of vegetable gardens and usual physical activities that can be carried out in limited spaces would go a long way in addressing such barriers creating an enabling environment for behaviour change. CHWs should be equipped with potential locally suitable options to share with community members to overcome such barriers.

Among the key facilitators for intervention implementation was the availability of key inputs such as interheart screening forms, referral forms and health education leaflets. Weather challenges also required that CHWs were provided with personal protective equipment including gumboots, umbrellas and t-shirts which were good motivation avenues and supported their identification within communities. As with most previous programmes, training was a key facilitator of intervention implementation [21, 38, 42] as CHWs had not interfaced with CVD interventions previously [18] supporting their understanding and contextualization of the disease, its risk factors and intervention [22]. The CHWs training programme was both didactic and experiential allowing CHWs to obtain the theoretical knowledge and field experience while receiving important feedback to improve, which was beneficial. Relatedly, during intervention implementation, frequent engagement with CHWs and goal setting helped to keep them on track and support supervision motivated them. The role of support supervision in CHW programmes has been well documented previously [21, 41, 42, 44, 47] and a strong component of this is required for successful programmes. Community structures such as local and religious leaders and local saving groups were key facilitators of intervention as they supported mobilisation of the community and/or were avenues through which members could be reached which CHWs took advantage of. As key components of a community health system, activating community interventions requires unlocking the potential of all community structures as a springboard for self-care interventions [15]. Another key advantage of the CVD prevention intervention was its outreach to build health facility capacity to deal with CVDs. Indeed, as had been anticipated [18], a functional health facility with available and friendly staff, drugs and equipment was a very important facilitator of the intervention. In their review of barriers and facilitators of CHWs engaging in NCD prevention and control in Asian countries, the integration of the health system with community CVD prevention interventions was key [21, 35]. Thus, a strengthened health system is required for the effective functionality of a community intervention.

Exploring the acceptability of the proposed intervention among the community and CHWs was an important step in understanding the community dynamics, opportunity costs, and anticipated barriers and facilitators which guided intervention refinement and delivery [18]. Indeed, some of the identified gaps were bridged prior to intervention implementation; however, other gaps were external and affected implementation. Based on the lessons from the acceptability part of our study [18] and this exploration of barriers and facilitators, future community programmes should consider exploring the prospective acceptability [48] of interventions and use findings to guide intervention implementation. Moreover, during intervention implementation, it is important to continuously engage with CHWs and local stakeholders to generate contextually relevant and innovative adjustments to resolve some of the impediments faced.

In this study, through using CFIR, we elicited key barriers and facilitators that spanned across the framework constructs thus furthering our understanding of the implementation process of a community-based CVD prevention programme led by CHWs. The framework also guided results presentation and comprehensive synthesis highlighting key elements that would otherwise have been missed and allowing for cross setting evaluation and comparison in this international project forming a key strength of this study. In addition, the study conducted a continuous process evaluation supporting timely reporting and discussion of barriers and facilitators and this together with the focus group discussions held at the end of the implementation cycle formed the study data enabling stepwise examination of the process and triangulation of responses. Moreover, all CHWs participated in the process evaluations and focus group discussions. With the increasing use of CFIR, there is an opportunity for comparison of findings across studies. As opposed to most previous studies that examined barriers and facilitators post- or pre-intervention [49], this study provides information on drivers of implementation as programme implementation is ongoing. Thus, it was not possible to compare performance across cases, determine strength of given implementation drivers or highlight magnitude of distinguishing parameters. Among the study weaknesses was the possibility of CHWs providing desirable responses due to their involvement in the study and their community-based supervisors being present during the discussions as note takers. However, this was unlikely as CHWs were usually upfront about the barriers they faced, and the support they felt they needed and efforts were further undertaken to re-assure CHWs to share any feedback so that the team was better able to support them and that their views would not influence their continued engagement in the programme. This study did also not obtain views from community members and other key stakeholders such as programme managers which would have helped in triangulation of CHW responses. This study contributes important information regarding the implementation process of a community CVD prevention intervention which should inform other programmes especially in low-income contexts.

## Conclusion

The community CVD prevention programmes showed promise within the context amidst implementation barriers and facilitators organised as per the CFIR. Indeed, factors such intervention complexity, cultural compatibility, enabling environment for behavioural change and CHW factors such as their demographics and competing demands require significant attention especially during intervention planning and implementation. On the other hand, strengthening programme facilitators including availability of inputs and protective equipment, thorough training of CHWs, working with community structures, frequent support supervision and engagements, CHW attributes such as motivation and commitment, and improving access to quality health services is important for successful implementation. These drivers of implementation should inform the design of impactful, scalable and sustainable CHW programmes for non-communicable diseases prevention and control.

## Supplementary Information

**Additional file 1.** FGD Discussion Guide.

**Additional file 2.** Consolidated criteria for reporting qualitative studies (COREQ): 32-item checklist.

## Data Availability

The data/transcripts used during the current study are available from the corresponding author on reasonable request.

## Abbreviations

CHWs: Community Health Workers
CVD: Cardiovascular disease
FGD: Focus Group Discussion
NCDs: Non-communicable disease
SPICES: Scaling-up Packages of Interventions for Cardiovascular disease prevention in selected sites in Europe and sub-Saharan Africa
WHO: World Health Organization
