# Supplementary material for: Barriers and facilitators of implementation of a community cardiovascular disease prevention programme in Mukono and Buikwe districts in Uganda using the Consolidated Framework for Implementation Research
Source: Implement Sci. 2020 Dec 9;15:106. doi: 10.1186/s13012-020-01065-0 (PMC7726905; doi:10.1186/s13012-020-01065-0)
Supplement: Supplementary file 1 — Additional file 1. FGD Discussion Guide. [file 13012_2020_1065_MOESM1_ESM.docx]

**Barriers and facilitators in implementation of a community cardiovascular disease prevention programme in Mukono and Buikwe districts in Uganda using the Consolidated Framework for Implementation Research**

Focus group discussion guide for community health workers

**General information**

District: __________________________ Sub county: _____________________________

Parish: ___________________________ Village: ________________________________

Date: ____________________________ Name of note taker: _______________________

FGD number: _______________ Number of FGD participants: ________________________

Starting time: ______________________ End time: _______________________________

**Brief instructions for the FGD facilitator/moderator:**

**Instructions to interviewer:**

- This discussion guide shouldn’t be followed word-for-word like a questionnaire, rather it should guide your discussion with the participants and ensure that all topics are covered.
- These interviews should be open-ended, with the participants’ responses determining the direction of the discussion. Be flexible. The discussion is expected to last about an hour.
- Start by building rapport with respondents, introductions and seek written consent for the discussion from each participant. Also discuss taking notes; seek oral consent for using recorder.
- Set and agree on ground rules.
- Record number assigned to each CHW, age, sex, education level and duration in CHW work at the end.

Questions

1. What is your understanding of your role in the project?
2. Briefly describe to us what you have been doing in the communities? What activities are you implementing? How are you implementing them? (probes: health education, using interheart tool, referral, collecting data, mobilizing other stakeholders like local and religious leaders)
3. How are the activities being received by the community? What do they say? What do they know about SPICES? How about the health facilities, how are they responding?
4. How would you describe your current capacity to implement the SPICES interventions? What are you able to do very well? Where are the gaps? What kind of knowledge/skills are available and what competences need to be developed further? What is your perception of the quality of the supporting materials, packaging, and bundling of the intervention for implementation? Is the intervention tailored to your context and practical to carry-out? What kind of extra support could you benefit from?
5. What are the barriers and enablers of the CVD prevention program? What kinds of changes or alterations have you needed to make to the program so it will work effectively? Who is involved in the implementation process? What barriers do the community members face in participating in the CVD program?
6. How are you finding it integrating CVD prevention activities into your usual activities? How is it working so far?
7. What factors are facilitating you to be able to implement the project interventions? How?
8. What challenges are you facing in carrying out the project interventions within your communities? (probes: time, uncooperative community members, motivation/incentives)
9. How is the support that the SPICES project is providing you? What could be done differently?
10. How could the SPICES program be improved?

*Note: Record the key characteristics of the CHWs (age, village, education level, occupation, years working as CHW)*

***Thank you for your time***
